# Recognition new energy vehicles based on improved YOLOv5

**DOI:** 10.3389/fnbot.2023.1226125

**Published:** 2023-07-28

**Authors:** Yannan Hu, Mingming Kong, Mingsheng Zhou, Zhanbo Sun

**Affiliations:** ^1^School of Computer and Software Engineering, Xihua University, Chengdu, Sichuan, China; ^2^School of Transportation and Logistics, Southwest Jiaotong University, Chengdu, Sichuan, China

**Keywords:** Intelligent Traffic Systems, new energy vehicle, traffic flow statistics, detect vehicles, license plate

## Abstract

In the field of Intelligent Traffic Systems (ITS), vehicle recognition is a hot research topic. Although different types of vehicles can already be recognized, further identification and statistics of new energy and fuel vehicles in unknown and complex environments remain a challenging task. In this paper, we propose a New Energy Vehicle Recognition and Traffic Flow Statistics (NEVTS) approach. Specifically, we first utilized the You Only Look Once v5 (YOLOv5) algorithm to detect vehicles in the target area, in which we applied Task-Specific Context Decoupling (TSCODE) to decouple the prediction and classification tasks of YOLOv5. This approach significantly enhanced the performance of vehicle detection. Then, track them upon detection. Finally, we use the YOLOv5 algorithm to locate and classify the color of license plates. Green license plates indicate new energy vehicles, while non-green license plates indicate fuel vehicles, which can accurately and efficiently calculate the number of new energy vehicles. The effectiveness of the proposed NEVTS in recognizing new energy vehicles and traffic flow statistics is demonstrated by experimental results. Not only can NEVTS be applied to the recognition of new energy vehicles and traffic flow statistics, but it can also be further employed for traffic timing pattern extraction and traffic situation monitoring and management.

## 1. Introduction

New energy vehicles significantly reduce tailpipe emissions by using electricity or other renewable resources to replace the fossil fuels in traditional vehicles, thereby contributing to the achievement of carbon reduction targets. Therefore, accurate monitoring and recognition of new energy vehicles are particularly important and a hot research topic of ITS (Zhao, 2023). In this paper, our objective is to address the issue of recognizing new energy vehicles and to conduct traffic flow statistics, using computer vision-based object detection techniques.

Traditional traffic flow statistics methods, such as magnetic coils and microwaves, have limitations in accuracy and applicability. Magnetic coil detection methods can easily lead to false positives when vehicles are closely spaced, and cannot identify vehicle types. In addition, magnetic coil equipment is susceptible to external environmental factors (Hata et al., 2019) such as road construction and weather changes, further reducing detection accuracy. Microwave detection experiences a certain degree of precision loss in congested traffic or when large vehicles are present, leading to inaccurate data. Moreover, installing microwave detection equipment is difficult and expensive (Ho and Chung, 2016), requiring destructive road modifications and increasing equipment and maintenance costs. Computer vision-based statistical methods can greatly improve efficiency and reduce costs. In recent years, there has been a significant proliferation of deep learning algorithms and their applications across diverse domains. For instance, in the field of object detection, where several algorithms are based on self-powered sensors (Huang et al., 2022; Yun et al., 2022b) and real-time detection (Yun et al., 2022a). Additionally, some methods apply object detection techniques to vehicle recognition and traffic flow statistics. In 2019, a two-stage Faster Region-based Convolutional Neural Network (Faster R-CNN) algorithm for vehicle recognition, represents decent detection results (Zhao and Li, 2019). However, the real-time requirements were not satisfied due to the sluggishness of the algorithm's detection speed. In 2020, a laser radar system combined with color camera technology was proposed to recognize vehicle objects (Yu et al., 2020). The cost is also very high because the algorithm needs lidar systems. Traditional methods can be used for traffic flow statistics, but there are limitations in vehicle identification and statistics. For example, magnetic coils and microwave detection methods cannot identify vehicle types and are susceptible to external environmental factors. In addition, these methods are expensive, requiring road modifications and maintenance. Computer vision-based methods for vehicle detection have addressed these limitations. However, current methods can only recognize different vehicle types, without further distinguishing and statistics on new energy vehicles and fuel vehicles. To solve these problems, we propose the New Energy Vehicles Recognition and Traffic Flow Statistics approach (NEVTS). Firstly, the vehicles are detected, and then their license plate colors are classified. Finally, the vehicles are identified as new energy vehicles or fuel vehicles based on their license plate colors, with green license plates representing new energy vehicles and non-green ones representing traditional fuel vehicles.

**Figure 1:**
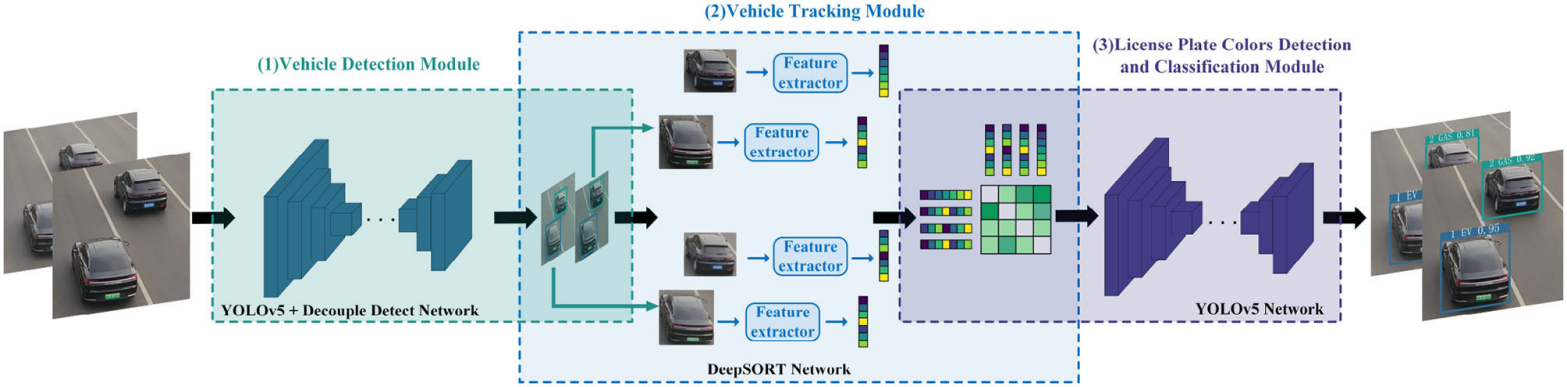
The overall framework of NEVTS. It contains three parts: (1) vehicle detection module, (2) vehicle tracking module, (3) license plate colors detection and classification module.

The overall architecture of the NEVTS model is depicted in Figure 1, which comprises three modules: vehicle detection module, vehicle tracking module, and license plate colors detection and classification module. The vehicle detection module is responsible for vehicle identification. Once a vehicle is identified, its position information is immediately transmitted to the vehicle tracking module. The vehicle tracking module tracks the vehicle based on the received information, then establishes a vehicle connection between two frames of the video. The license plate colors detection and classification module locates and detects the license plate color when a collision occurs between the vehicle and a virtual line segment, green for new energy vehicles. Then, traffic flow is calculated based on the vehicle's trajectory and position. This method enables more accurate and efficient statistics for new energy vehicles, real-time performance, and recognition efficiency.

Overall, our contributions are as follows:

- We improve vehicle detection and localization performance by decoupling YOLOv5 prediction and classification tasks.
- We proposed an approach that, based on the recognition of vehicle type, further detects and classifies the color of the vehicle's license plate to achieve the recognition of new energy vehicles.
- Based on NEVTS, we perform statistics on the flow of new energy vehicles in different scenarios, and experiments demonstrate the real-time performance and accuracy of our method.

The rest of this paper is organized as follows. Section 2 introduces the exploration of computer vision-based object detection and related vehicle tracking in related work. Following that, Section 3 outlines the proposed NEVTS approach in this study. In Section 4, two datasets and the obtained experimental results for new energy vehicle traffic flow statistics are presented. A conclusion is drawn in Section 5.

## 2. Related work

To achieve real-time tracking of vehicles and traffic flow counting, we have explored computer vision-based object detection algorithms and related vehicle tracking algorithms.

### 2.1. Object detection analysis

The classification of existing computer vision-based object detection algorithms primarily falls into two categories (Arya et al., 2021): two-stage detection algorithms, such as R-CNN, SPP-Net, Fast R-CNN, Faster R-CNN, and FPN, and one-stage detection algorithms, including the YOLO series, SSD, and RetinaNet.

Selective search is used by R-CNN (Girshick et al., 2014) to generate 2000 candidate boxes that are most likely to contain objects and then extract features from these candidate boxes. However, because R-CNN extracts deep features from each candidate box separately, so it has a slow inference speed. To mitigate this concern, the initial step involves extracting comprehensive image features through feature sharing. Subsequently, the spatial pyramid pooling (SPP) (He et al., 2015) operation, commonly referred to as SPP-Net, is used. Fast R-CNN (Girshick, 2015) combines the characteristics of R-CNN and SPP-Net by initially extracting profound image features, followed by the utilization of region of interest (RoI) pooling operation. In 2015, the Faster R-CNN algorithm (Ren et al., 2015) was proposed, which uses the region proposal network (RPN) for the first time to replace the selective search algorithm. To cope with changes in object scale, the FPN detection algorithm (Lin et al., 2017a), proposed subsequently, enhances the capabilities of Faster R-CNN by introducing multiple layers of features and feature fusion mechanisms. Compared with two-stage algorithms, which require generating region proposals, single-stage algorithms directly regress the position and class of the target. As a result, they offer faster speed and a simpler structure. In 2016, the YOLOv1 algorithm (Redmon et al., 2016) was initially suggested by inputting the object picture into the neural network to acquire the object's bounding box and classification results directly. In 2017, the YOLOv2 algorithm (Redmon and Farhadi, 2017) was introduced to tackle the problem of weak detection precision in YOLOv1. This was done by including Batch Normalization (BN) (Ioffe and Szegedy, 2015) post each convolutional layer, utilizing multi-scale training methods for enhanced object detection, and refining a high-resolution dataset with a pre-trained Convolutional Neural Network (CNN). The YOLOv3 algorithm (Redmon and Farhadi, 2018) was brought in 2018. It incorporates the residual module and 9 anchor boxes to facilitate detection while maintaining speed and enhancing the detection precision. In 2020, the YOLOv4 algorithm (Bochkovskiy et al., 2020) is proposed. It uses a better Mish (Misra, 2019) activation function and introduces the SPP module to further improve detection. The YOLOv5 algorithm was proposed almost simultaneously with the YOLOv4 algorithm, which introduces mosaic data enhancement, adaptive anchor, and focus structure to further improve detection accuracy. The YOLOv5 has the feature of a smaller and simpler model. YOLOvX (Ge et al., 2021) introduces a decoupled detection head, but the inputs for the regression and localization tasks in the decoupled head still come from a single head. YOLOv7 (Wang et al., 2023) proposes a training method for an auxiliary head, which aims to improve accuracy by increasing training costs. YOLOv8 uses an anchor-free mechanism and improves the decoupled head, but the inputs for the decoupled head still come from a single head. In 2016, the SSD algorithm (Liu et al., 2016) was proposed, which leverages feature maps from various layers to detect objects of diverse scales. Feature maps with higher resolutions in the front detect smaller objects, while feature maps with lower resolutions in the back detect larger objects. In 2017, Lin et al. (Lin et al., 2017b) proposed RetinaNet, which introduces a focal loss function to reconstruct the standard cross-entropy loss function, making the detector more focused on classifying difficult samples during the training process.

### 2.2. Object tracking analysis

According to the quantity of tracked objects, object tracking methods are categorized into two types, single-object tracking, and multi-object tracking (Deori and Thounaojam, 2014). Single-object tracking refers to detecting a single object in successive frames and then predicting its size and position in subsequent frames. Typical single-object tracking algorithms include Mean Shift (Comaniciu and Meer, 2002), Tracking-Learning-Detection (TLD) (Kalal et al., 2011), Kernelized Correlation Filter (KCF) (Henriques et al., 2014), etc. Multi-object tracking tracks the size and position of multiple objects. Typical multi-object tracking algorithms include SORT (Bewley et al., 2016) and DeepSORT (Wojke et al., 2017), etc.

To achieve temporal tracking of vehicles, a majority of vehicle tracking methods are based on a fundamental principle of judging whether two vehicles in adjacent frames are identical by utilizing spatial distance (Koller et al., 1992; Deori and Thounaojam, 2014). These methods can be classified into the following four categories:

- Model-based methods: The core of this method is the creation of an accurate two-dimensional model of known vehicle objects. Then, the acquired images are aligned with this model (Koller et al., 1992). The approach is not suitable for real-time processing due to its computational complexity, which requires a large amount of computation.
- Region-based methods: This method tracks vehicles in the time domain. The detection module identifies individual pixel-connected blocks representing the vehicles (Deori and Thounaojam, 2014). This approach performs better in sparse vehicle scenarios.
- Dynamic contour-based method: First, the vehicle contour is drawn. It is then updated in the following frames to accomplish the goal of tracking (Deori and Thounaojam, 2014). Nonetheless, the effectiveness of the approach deteriorates significantly in crowded road conditions. This is because shadows between vehicles turn several adjacent connection blocks into a single block. This results in missed detections and false positives.
- Feature-based methods: Vehicle features are treated as minimal tracking units, including points, lines, and curves (Deori and Thounaojam, 2014). The advantage of this method is that even if the vehicles block each other, a large part of their features is still visible.

In this paper, the DeepSORT algorithm is used for vehicle tracking. It belongs to Feature-based methods.

## 3. Method

**Figure 2:**
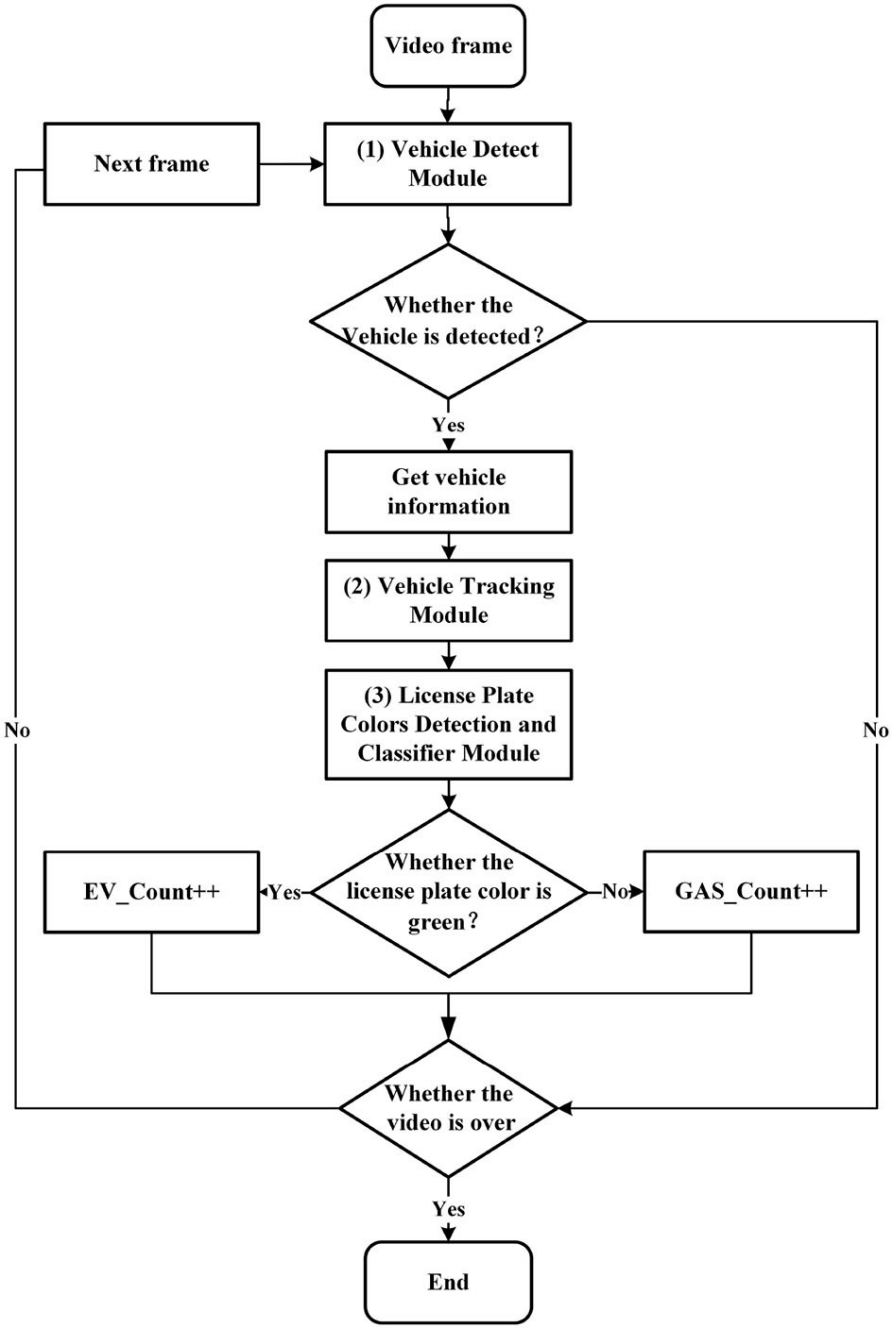
The process of NEVTS. EV_Count represents the number of new energy vehicles. GAS_Count represents the number of fuel vehicles. When the detected license plate is green, the EV_Count number is increased by one, non-green GAS_Count number is increased by one.

A detailed presentation of the NEVTS is provided in this section. As shown in Figure 2, three constituents make up the approach: vehicle detection module, vehicle tracking module, and license plate colors detection and classification module. First, the vehicle detection module monitors the video image frame to identify the presence of vehicles using an improved YOLOv5 algorithm. Then, the vehicle tracking module receives the vehicle location information, confidence, and category information (car, bus, truck) from the vehicle detection module to enable real-time vehicle tracking through the DeepSORT algorithm. Finally, When the tracked vehicle intersects with the predefined virtual line segment, the YOLOv5 algorithm is used to detect the license plate position and color, and distinguish new energy vehicles and fuel vehicles, followed by statistics of traffic flow.

**Figure 3:**
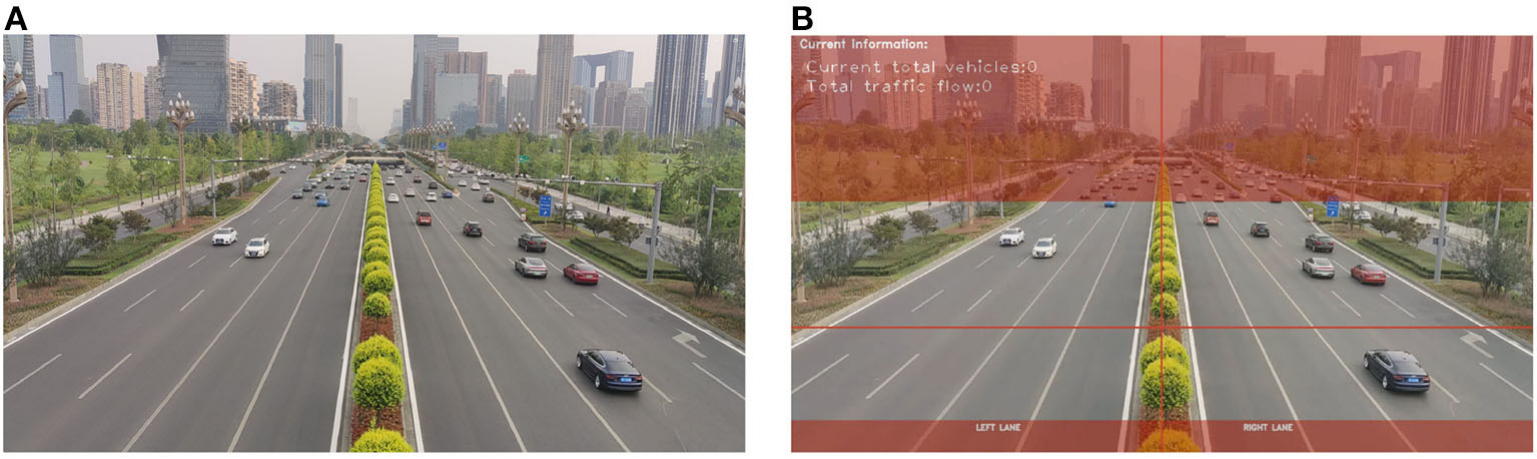
Image detection regions configuration. **(A)** The original image. **(B)** The image after setting the detection area.

We obtain videos from cameras under various weather conditions and decode them. Subsequently, we segment the detection regions in the videos. As shown in Figure 3, the detection regions are predetermined. The camera angles are approximately the same and the captured image scenes are very similar. In case of changes in the angle of the video, we manually adjust the corresponding detection regions (non-red regions). The detection video images are applied with a mask, where the red region mainly comprises areas far from the camera. To reduce processing time and improve recognition accuracy, we only detect and track vehicles in non-red areas.

### 3.1. Vehicle detection module

To provide vehicle location and confidence information to the vehicle tracking module, we first detect the vehicles within the image monitor areas. Then, we label the detected vehicles. Finally, we obtain the location and confidence of the detected vehicles. To achieve more accurate positioning of vehicles in crowded conditions, we apply TSCODE (Zhuang et al., 2023) to decouple the prediction and classification tasks of YOLOv5. The algorithm is enhanced to improve vehicle detection performance.

**Figure 4:**
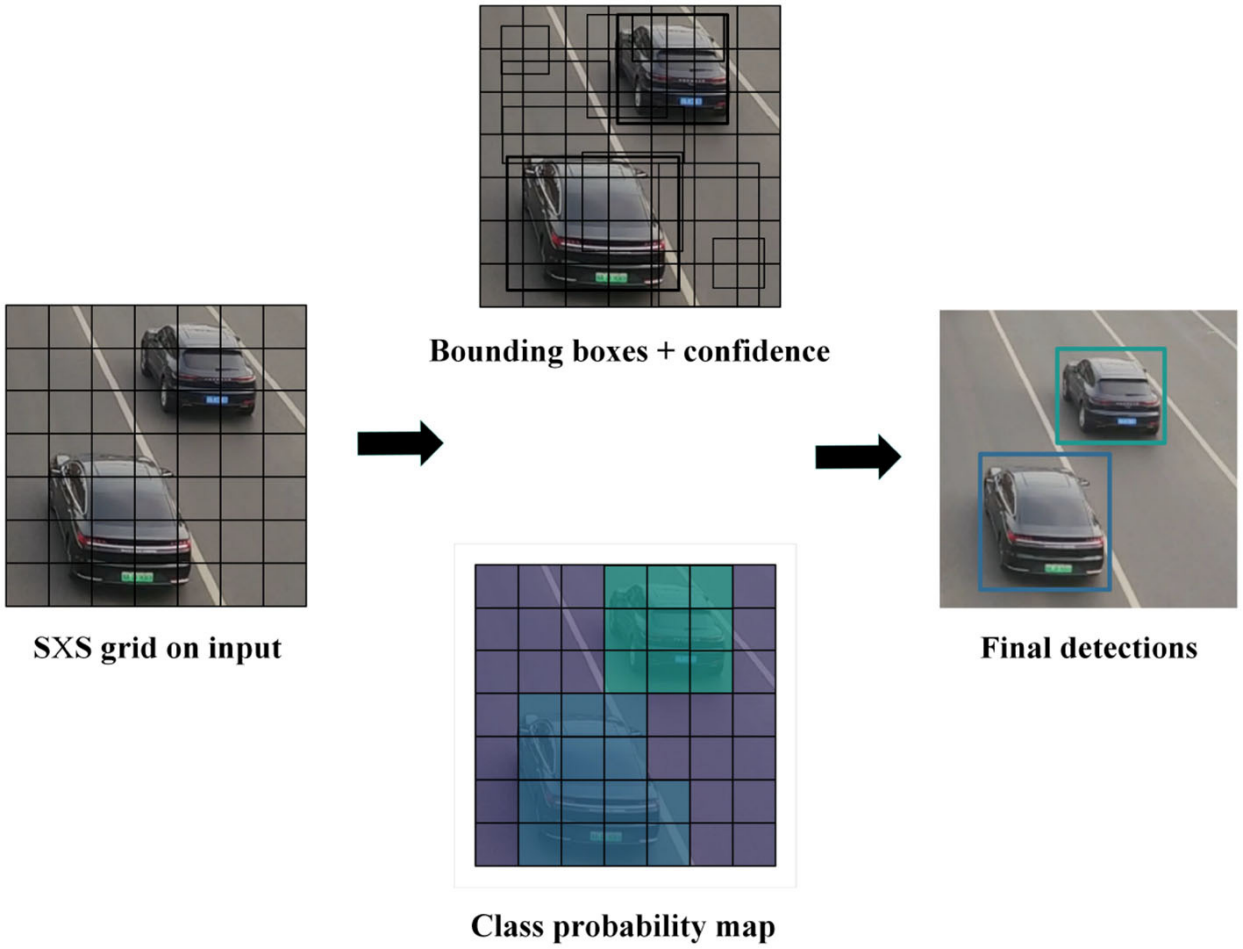
The YOLOv5 vehicle detection algorithm process. YOLOv5 performs feature extraction on input images using a feature extraction network and predicts the position and category information of the vehicle in the image to achieve vehicle detection.

As shown in Figure 4, when detecting vehicles, features are extracted on the input images using a feature extraction network. If there are C classes, dividing the image into grid cells of size S×S results in (5 + C) attribute values for each grid cell (Zhang et al., 2022). The first four values represent the coordinates of the detection box, the fifth value represents the objectness score, and the remaining values represent the confidence scores of the C classes. We set the confidence threshold to 0.75, so only boxes with confidence scores > 0.75 and their corresponding class names are displayed. However, there may be the issue of multiple detection boxes for the same object. To address this issue, we use Non-Maximum Suppression (NMS) (Neubeck and Van Gool, 2006). First, the box with the highest confidence score is selected. Next, remove any overlapping boxes with an Intersection-Over-Union (IOU) threshold >0.25. This process ensures that each object has only one detection box.

**Figure 5:**
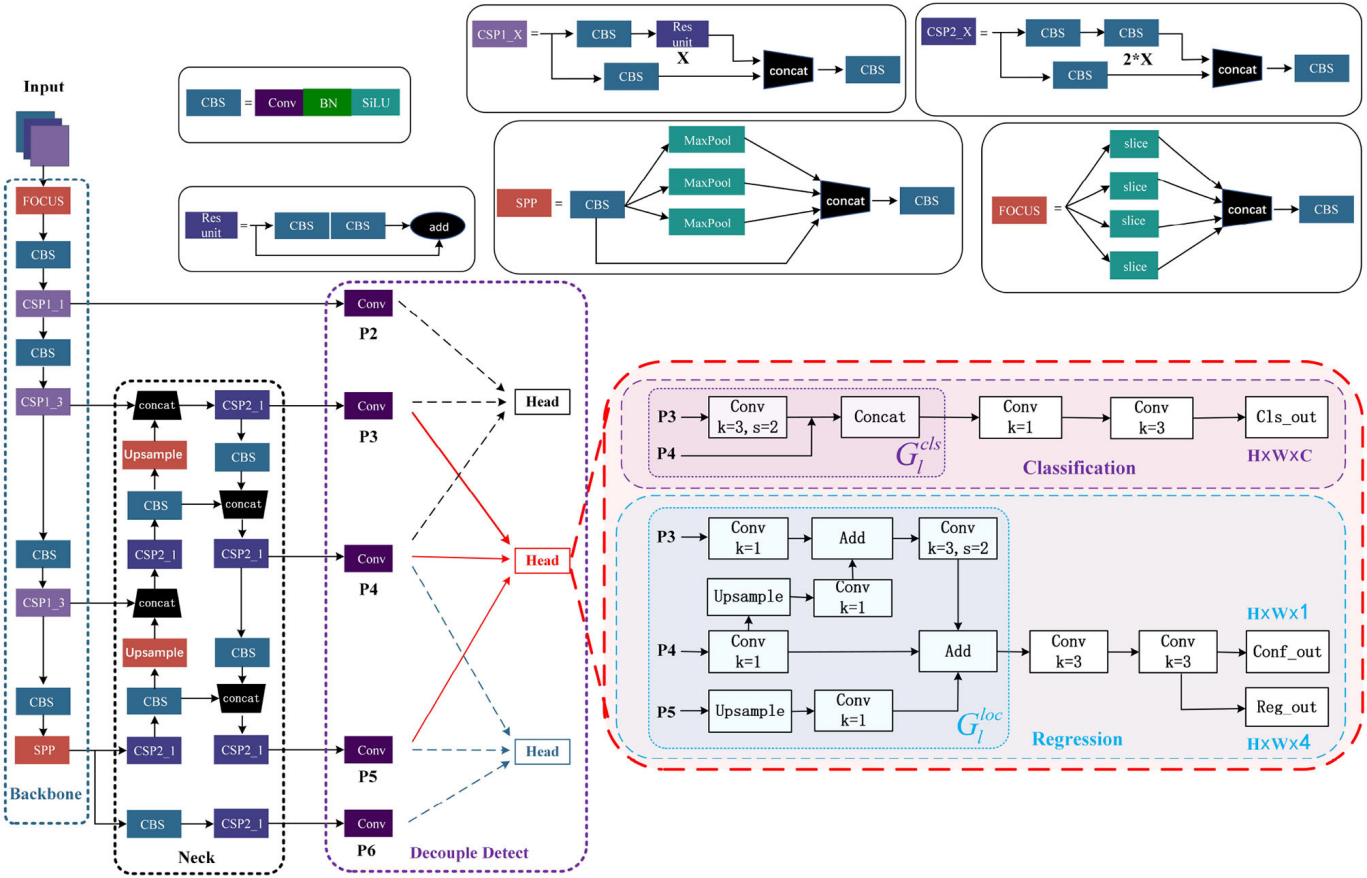
The overall network architecture of YOLOv5 after decoupling. Two new output layers, P2 and P6, are introduced. Every three adjacent layers form a new head.

Figure 5 shows that the YOLOv5 model comprises of four main constituents, namely input, neck, backbone, and detect. We have improved the Detect component. TSCODE is applied to decouple Detect.

#### 3.1.1. Decouple Detect

In the domain of object detection, the primary sub-tasks are classification and localization, which show different preferences for feature context (Zhuang et al., 2023). The classification task has a coarser granularity and prioritizes semantic information, while localization has a finer granularity and seeks to capture boundary or texture features. The YOLOv5 algorithm uses a separation header to learn different feature contexts for each task, but the input features of these heads still come from the same layer, which leads to an imperfect balance between classification and localization. The TSCODE generates feature codes that are spatially rough but semantically strong for the classification branch. For the localization branch, high-resolution feature mapping with more edge information is provided to better regress object boundaries. Originally, YOLOv5 has only three output layers: P3, P4, and P5. To truly decouple the detection process, two new output layers, P2 and P6, are introduced. Every three adjacent layers form a new head. For example, P3, P4, and P5 form one Head.

Leverages the feature maps from two levels, i.e., *P*_*l*_ and *P*_*l*+1_, to generate a semantically rich feature map for classification. First, downsample *P*_*l*_ by a factor of 2 and then concatenate it with *P*_*l*+1_ to generate the final $G_{l}^{cls}$. Finally, feed $G_{l}^{cls}$ into the classification function. The $G_{l}^{cls}$ can be written as


(1) $$\begin{array}{l} \begin{array}{c} G_{l}^{cls}=\mathrm{Concat}\left(\mathrm{DConv}\left(P_{l}\right),P_{l+1}\right) \end{array} \end{array}$$

where Concat(·) and DConv(·) represent concatenation and a shared downsampling convolutional layer.

For computational efficiency, adopt a simplistic U-Net to fuse *P*_*l*−1_, *P*_*l*_ and *P*_*l*+1_. *P*_*l*_ is first upsampled by a factor of 2 and then aggravated with *P*_*l*−1_. A stride of 2 is used for a 3 × 3 convolutional layer and downsamples it to the resolution of *P*_*l*_. This design effectively preserves the detail information in *P*_*l*−1_. At last, *P*_*l*+1_ is upsampled and aggregated to generate the final $G_{l}^{loc}$. To achieve proper feature map aggregation, a 1 × 1 convolutional layer is utilized in the middle of the process. Finally, feed $G_{l}^{loc}$ into the regression function. The $G_{l}^{loc}$ can be written as


(2) $$G_{l}^{loc}=P_{l}+\mu \left(P_{l+1}\right)+\mathrm{DConv}\left(\mu \left(P_{l}\right)+P_{l-1}\right)$$

where μ(·) represents upsample.

### 3.2. Vehicle tracking module

To implement the tracking of vehicles, we use the DeepSORT algorithm, which matches detected vehicles in two consecutive frames of a video and assigns a unique ID number to each vehicle to determine the vehicle's motion trajectory. If the vehicle ID isn't present in the ID list and the previous frame, it is a new vehicle. The new vehicle is initialized as a fuel vehicle and assigned a new unique ID. If a vehicle ID present in the previous frame or ID list is not detected in the current frame, it may have left the monitor area. To improve accuracy, a dual threshold is generally applied to determine whether the vehicle has truly left the monitor area. The two thresholds are as follows: if the vehicle is not detected for 25 consecutive frames or 1 s, it is considered to have left the monitor area.

**Figure 6:**
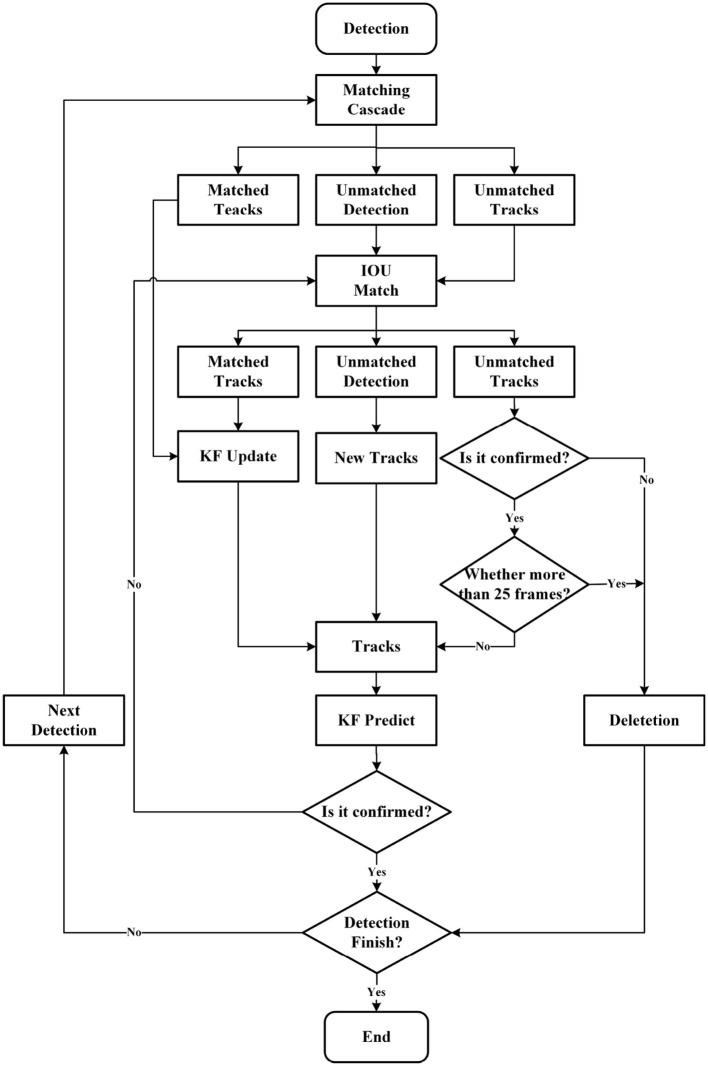
The process of DeepSORT object tracking. Matching Cascade and IOU Match are both used to match and compute the detection boxes in the current frame with the prediction boxes from the previous frame (tracks). Their matching results can be divided into three types: prediction box and detection box match (matched tracks), detection box miss in the current frame (unmatched detection), and prediction box miss in the previous frame (unmatched tracks).

The process of DeepSORT object tracking is illustrated in Figure 6. To be specific, first, the original frames of the video are obtained. Then, the YOLOv5 algorithm is used to detect objects and obtain the object detection boxes. Next, all the object vehicles corresponding to the object boxes are cropped out, their features are extracted, and to update and predict vehicle information, the Kalman Filter (KF) (Hamuda et al., 2018; Welch, 2020) is adopted. Afterward, similarity calculations are performed to determine the matching degree between the vehicles in consecutive frames using the Hungarian algorithm (Hamuda et al., 2018). Finally, data association is carried out to assign an ID to each vehicle.

#### 3.2.1. Hungarian algorithm

To associate a tracking object with detection boxes, we first utilize KF to predict each tracking object's movement. We then calculate its similarity to all the detection boxes. Finally, the Hungarian algorithm is applied to associate each tracking object with the best-matched detection box and update its state. When using the Hungarian algorithm for object association, Mahalanobis distance is used for the similarity calculation method. Because Mahalanobis distance is a distance measurement based on the covariance matrix, it can accurately reflect the differences between data by considering the correlation and covariance between data. The Mahalanobis distance formula is


(3) $$D(j,k)={\left(a_{k}-b_{j}\right)}^{T}S_{j}^{-1}\left(a_{k}-b_{j}\right)$$

where *D*(*j, k*) is the Mahalanobis distance between ***a***_*k*_ and ***b***_*j*_, ***a***_*k*_ and ***b***_*j*_ represent measured and predicted distributions, respectively. *S*_*j*_ is the matrix of the covariance between the two distributions, which is a real symmetric positive definite matrix. $S_{j}^{-1}$ is the inverse matrix of S_*j*_.

#### 3.2.2. Kalman Filter algorithm

The KF is a state estimation algorithm that combines prior knowledge with measurement updates and has two stages.

The first stage is mainly prediction. By utilizing the previous covariance matrix and state, KF estimates the current object's state and covariance matrix. The predicted position is then determined based on the estimated state. Specifically, according to the optimal estimated value ${\hat{x}}_{t-1}$ at time *t* − 1, the optimal estimated value ${\hat{x}}_{t}^{-}$ (predicted value) at time *t* is obtained, where *F* is the state transition matrix, *B* is the control matrix, and *u*_*t*−1_ is the control input.


(4) $${\hat{x}}_{t}^{-}=F{\hat{x}}_{t-1}+Bu_{t-1}$$

According to the covariance matrix *P*_*t*−1_ of the optimal estimation at time *t* − 1, the covariance matrix $P_{t}^{-}$ of the optimal estimation at time *t* is obtained, and *Q* is the covariance matrix of the engineering noise.


(5) $$P_{t}^{-}=FP_{t-1}F^{T}+Q$$

The second stage is mainly updating. KF incorporates the detection results to update the object's states and covariance matrix, which can refine the predicted position and enhance the tracking performance. To be specific, the KF gain *K*_*t*_ at the current time is obtained, where *H* is the observation matrix and *R* is the covariance matrix of the observation noise.


(6) $$K_{t}=P_{t}^{-}H^{T}{\left(HP_{t}^{-}H^{T}+R\right)}^{-1}$$

According to the estimated value ${\hat{x}}_{t}^{-}$ (predicted value) at time *t* and the observed value $K_{t}\left(z_{t}-H{\hat{x}}_{t}^{-}\right)$ at time *t*, the optimal estimate ${\hat{x}}_{t}$ is obtained, where *z*_*t*_ is the measured value.


(7) $${\hat{x}}_{t}={\hat{x}}_{t}^{-}+K_{t}(z_{t}-H{\hat{x}}_{t}^{-})$$

Continue to update the covariance matrix of the optimal estimation.


(8) $$P_{t}=(I-K_{t}H)P_{t}^{-}$$

Through the combined use of prediction and updating, KF can reduce the noise and uncertainty inherent in the system and enhance the robustness of the DeepSORT algorithm.

### 3.3. License plate colors detection and classification module

We aim to detect and classify the license plate colors of vehicles by location and vehicle ID information of the tracked vehicles. Furthermore, based on this information, we crop the tracked vehicle areas and employ the YOLOv5 algorithm for license plate detection. Specifically, first, the areas of the vehicle being tracked are detected, and identify areas that may contain license plates, such as the front and rear of the vehicle. Then, it divides each potential region of a license plate into multiple small cells and predicts the probability of each cell containing a license plate and the coordinates of the location of the license plate. Finally, we recognize the color of the license plate.

During vehicle tracking, first, we assume all vehicles to be fuel-based cars and set a virtual line segment in the detection area. Then, we only start the detection and classification of the license plate when a tracked vehicle collides with the virtual line segment. We classify vehicles into two categories: green license plates, and non-green license plates, with only green license plates, considered new energy vehicles and non-green license plates considered fuel-based vehicles. Finally, The traffic flow of new energy and fuel-based vehicles is calculated.

### 3.4. Loss function

The YOLOv5 object detection used in the vehicle detection module mainly involves three losses, including classes loss (*L*_*cls*_), objectness loss (*L*_*obj*_), and location loss (*L*_*loc*_) (Wu et al., 2021). Classes loss uses Binary CrossEntropy (BCE) Loss (Wu et al., 2021, 2022) and the calculation of the positive samples' classification loss is exclusively taken into account. However, *BCELoss* is still implemented for objectness loss to estimate the confidence loss of all samples. On the other hand, Complete-IoU (CIoU) Loss (Zheng et al., 2020) is leveraged to determine the location loss of only the positive samples. The overall *Loss* as follows, where λ_1_,λ_2_,λ_3_ is the balance coefficients.


(9) $$LOSS=\lambda_{1}L_{cls}+\lambda_{2}L_{obj}+\lambda_{3}L_{loc}$$

*BCELoss* as follows


(10) $$BCELoss=-w_{n}[y_{n}\cdot \log x_{n}+(1-y_{n})\cdot \log(1-x_{n})]$$

and *CIoULoss* is


(11) $$CIoULoss=1-IoU+\frac{\rho^{2}(b,b^{gt})}{c^{2}}+\alpha v$$

## 4. Experiment

This section delves into the experiments conducted. Firstly, we illustrate the formation of two public datasets and provide clarity on the experimental details. Subsequently, an account of the experimental protocol is given. Ablation studies and parameter analysis are performed, followed by a thorough analysis of the overall experimental results to demonstrate the effectiveness of our approach.

### 4.1. Dataset

#### 4.1.1. MS COCO and Pascal VOC dataset

To construct a high-quality vehicle detector, utilizing all classes present within datasets like the MS COCO (which has 80 classes) and Pascal VOC (which has 20 classes) is not the best option. Even though these datasets are often used in the training of object detection models, it is not an optimal choice for constructing an effective vehicle detection network. To address this issue, a combined dataset of cars, buses, and trucks was employed from both MS COCO and Pascal VOC datasets for the training process. The combined vehicle detection dataset comprises 17,019 training images and 1,452 testing images.

#### 4.1.2. CCPD dataset

The CCPD dataset is a large and diverse open-source dataset of carefully annotated Chinese urban license plates. It comprises two main subsets, namely the CCPD2019 dataset and the CCPD2020 (CCPD-Green) dataset. The CCPD2019 dataset contains only regular license plates, whereas the CCPD2020 dataset contains only new energy vehicle license plates (green) with 10,000 images. Each image in the CCPD dataset contains only one license plate, and the license plate province is predominantly “Anhui.” Despite lacking dedicated annotation files, each image in the CCPD dataset contains a wealth of annotation information, with the file name of each image corresponding to its data annotation.

### 4.2. Experimental details

In the experiments, the entire network is trained on a GPU (NVIDIA RTX 3090 24 GB) and a CPU (15 vCPU Intel(R) Xeon(R) Platinum 8350C CPU @ 2.60 GHz). In our method, the improved YOLOv5 network model is used to detect the vehicle. Meanwhile, we initialized the learning rate to 0.01, set the batch size to 64, set the epoch to 300, and set the iou_thres to 0.65. In the DeepSORT algorithm for vehicle tracking, The MAX_IOU_DISTANCE is set to 0.7, which allowed us to disregard associations with costs that exceeded the value.

### 4.3. Experimental protocol

To demonstrate the effectiveness of our approach in recognizing new energy vehicles and traffic flow statistics, three captured testing videos are used for the empirical evaluation of the method. Video1, video2, and video3 are three videos captured in day-time, high noon, and night-time scenes respectively, and they are both filmed on sections of the motorway. We compare the experimental results in each scene with the manually annotated truth results. *Precision* and *Recall* (Sajjadi et al., 2018) are used as evaluation metrics.

*Precision* represents the proportion of accurately detected vehicles to the total number of detected vehicles. A higher Precision indicates that the algorithm has fewer false detections and missed detections. Its formula is


(12) $$\begin{array}{l} \begin{array}{c} Precision=\frac{TP}{\left(TP+FP\right)} \end{array} \end{array}$$

where *TP* represents the number of vehicles correctly detected by the detector among all the actual vehicles present, and *FP* represents the number of non-existent vehicles incorrectly detected by the detector among all the non-existent vehicles.

*Recall* represents the proportion of correctly detected vehicles to the total number of actually present. A higher Recall indicates that the algorithm has fewer missed detections. Its formula is


(13) $$Precision=\frac{TP}{\left(TP+FP\right)}$$

where *FN* represents the number of vehicles that were not detected by the detector among all the actual vehicles present.

*F*1 is the harmonic mean of *Precision* and *Recall*, its formula is


(14) $$\begin{array}{l} \begin{array}{c} F1=\frac{2*Precision*Recall}{\left(Precision+Recall\right)} \end{array} \end{array}$$

**Figure 7:**
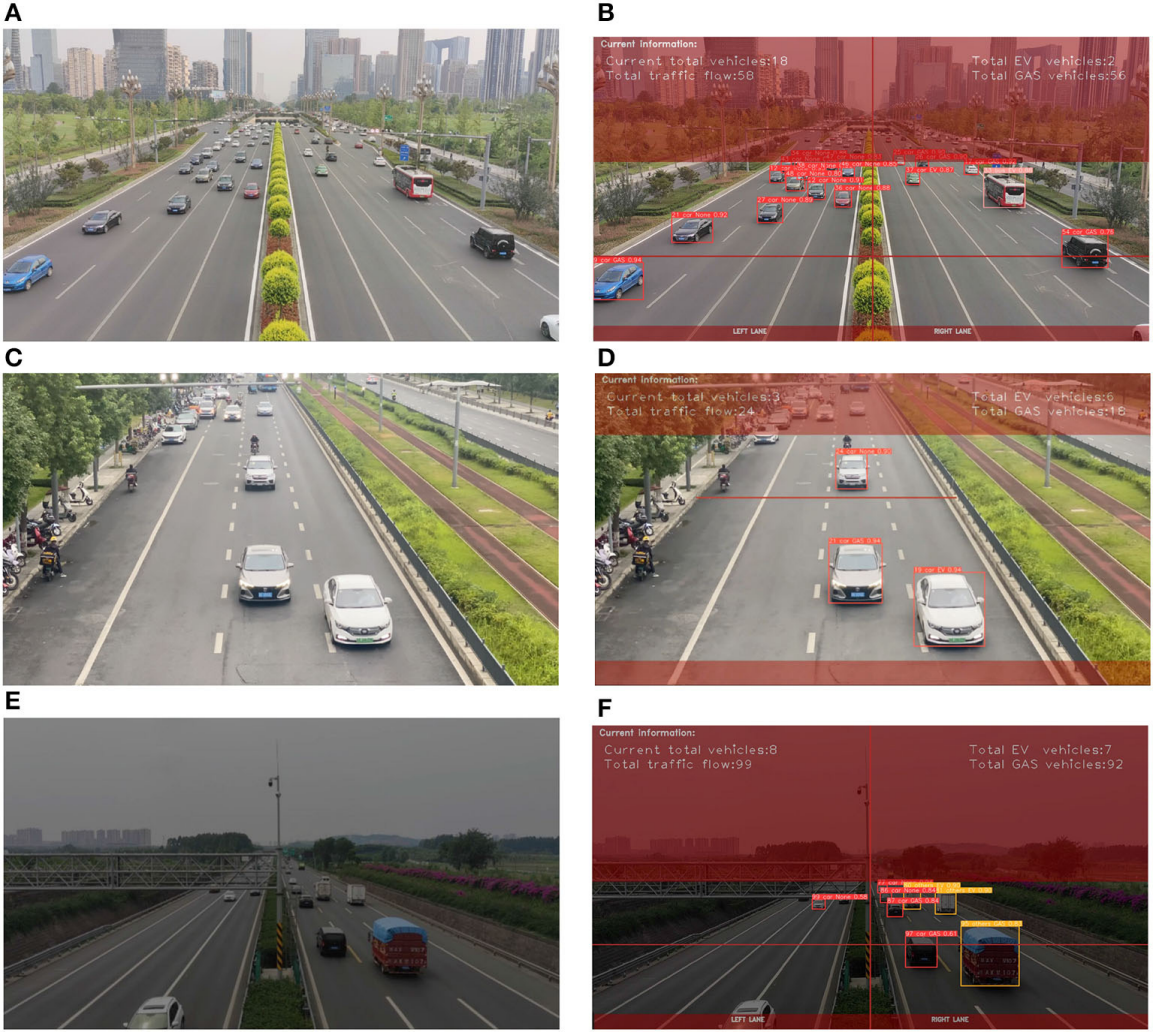
The experimental results with different lighting conditions: during day-time, high noon, and night-time. **(A)** Original day-time scene image. **(B)** Empirical evaluation of the day-time scene. **(C)** Original high noon scene image. **(D)** Empirical evaluation of high noon scene. **(E)** Original night-time scene image. **(F)** Empirical evaluation of the night-time scene.

The performance of the NEVTS approach is illustrated in Figure 7. The proposed approach exhibits high accuracy, as confirmed by experimental evaluations.

### 4.4. Ablation Studies

#### 4.4.1. Study of ablation

**Figure 8:**
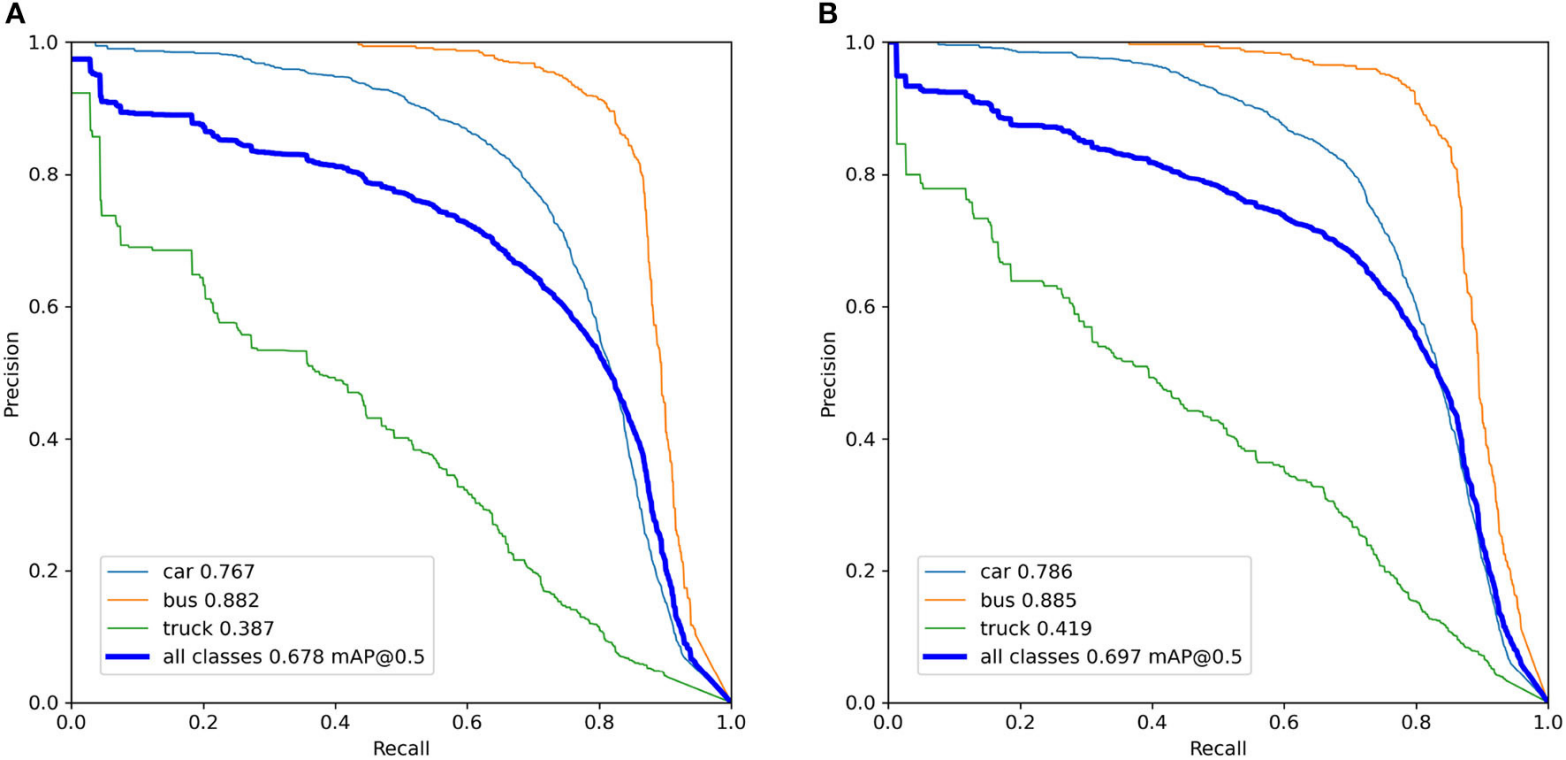
The precision-recall (PR) curves of the YOLOv5 algorithm with and without decoupled detection heads during training. **(A)** YOLOv5 algorithm without decoupled detection heads. **(B)** YOLOv5 algorithm with decoupled detection heads.

We conducted ablation studies to evaluate the efficacy of our design for detecting vehicles. Our study involved comparing the performance of the YOLOv5 algorithm with and without decoupling detection heads. Through comparative experiments, we validated the effectiveness of our method. The results of the ablation experiments are demonstrated in Table 1, which indicates an improvement in the mean Average Precision (mAP) (Henderson and Ferrari, 2017) metric. Furthermore, we visualize the Precision-Recall (PR) curves of the YOLOv5 algorithm with and without decoupled detection heads during training, as illustrated in Figure 8. After decoupling, the performance of all aspects improves, with mAP@0.5 for all classes increasing from 0.678 to 0.697.

**Table 1:** Experimental results on a composite dataset consisting of a combination of cars, buses, and trucks that were selected from the MS COCO and Pascal VOC datasets.

**Method**	**Class**	**Labels**	**P**	**R**	**mAP@0.5**	**mAP@.5:.95**
YOLOv5	Car	3105	77.2	70.4	76.7	51.3
	Bus	605	89.5	82.3	88.2	72.4
	Truck	415	43.1	47.0	38.7	25.6
	All	4125	70.0	66.6	67.8	49.3
YOLOv5 + decouple detect	Car	3105	**79.5**	**71.1**	**78.6**	**53.3**
	Bus	605	88.2	81.8	**88.5**	**73.5**
	Truck	415	**44.1**	**47.7**	**41.9**	**28.1**
	All	4125	**70.6**	**66.9**	**69.7**↑1.9	**51.6**↑2.3

P represents *Precision*. R represents *Recall*.

↑ represents performance upgrade. In bold are the best outcomes for each object category. The improved algorithm proposed is YOLOv5+Decouple Detect.

#### 4.4.2. Visual analysis

**Figure 9:**
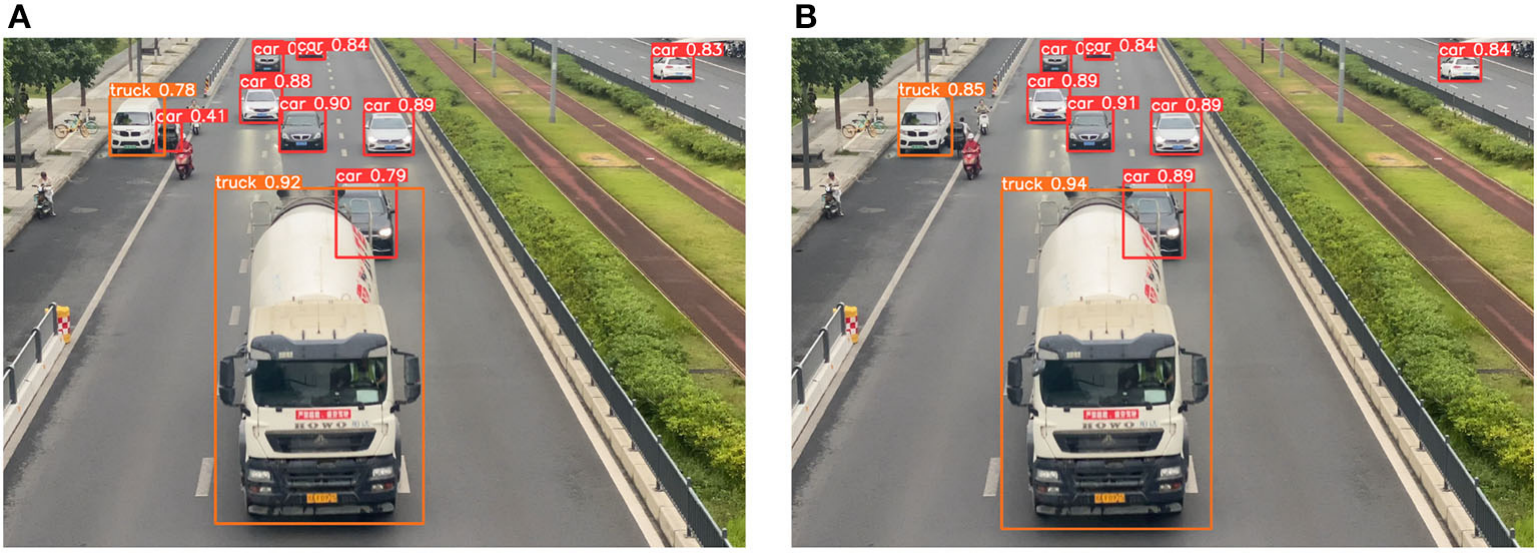
The YOLOv5 algorithm with and without decoupled heads detects results on the same image. **(A)** YOLOv5 algorithm without decoupled detection heads detects results. **(B)** YOLOv5 algorithm with decoupled detection heads detects results.

The YOLOv5 algorithm with and without decoupled detection heads detect results in this section are shown in Figure 9. Notably, the improved YOLOv5 algorithm exhibited superior performance in object localization, particularly in scenarios involving object occlusion. This enhancement has increased the accuracy of vehicle traffic counting.

### 4.5. Parameters analysis

**Figure 10:**
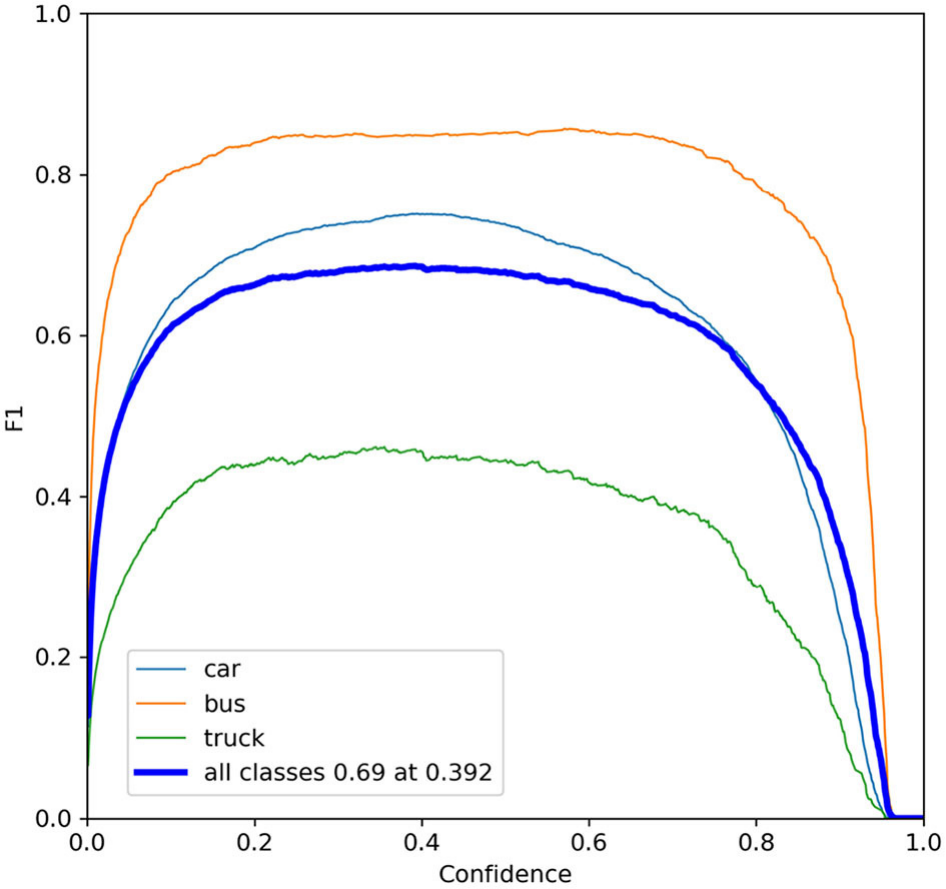
The F1-confidence curves.

Confidence mainly controls the threshold for vehicle detection. It indicates the extent to which the model detects vehicles in the image. Confidence can be used for filtering detection results and improvement of model accuracy. The F1-Confidence curve is shown in Figure 10. The vehicle detection performance is dependent on the Confidence value's magnitude. The best performance for car detection is achieved when Confidence is set between 0.4 and 0.6. The optimal performance for bus detection is attained when Confidence is set between 0.3 and 0.5. For truck detection, the optimal performance is achieved when Confidence is set between 0.3 and 0.4. The overall performance on all types of vehicles is best when Confidence is set to 0.392. Different confidence levels can be set depending on the scenario being used.

### 4.6. Experimental results and analysis

We have produced statistics on the experimental results, which are shown in Table 2. Our approach, NEVTS, achieved high performance in identifying new energy vehicles. The overall accuracy of NEVTS was evaluated using *Precision* and *Recall*, and compared with the actual results of manual labeling. Performance performed well in both daytime and night-time scenes, especially in daytime scenes, with a *Precision* of 0.923. The approach is based on the traffic scene, *Precision* and *Recall* is not the only evaluation standard, speed is another important parameter for the approach, and the traffic statistics approach achieved a speed of 31.69 fps, which meets the real-time requirements and maintains a certain level of accuracy. The NEVTS approach is capable of distinguishing vehicle types. Our proposed approach is that the accuracy of license plate recognition may decrease under exposure to intense sunlight or license plate blocking and late at night, as even humans may have difficulty discerning license plate colors under such conditions, but the vehicle can still be detected.

**Table 2:** Experimental statistics were obtained in day-time scene (video1) and high soon (video2) and night-time scene (video3) and compared with the actual results of manual labeling.

	**Vehicle_type**	**Video1**	**Video2**	**Video3**
NEVTS traffic statistics	EV_vehicler	39	14	15
	GAS_vehicle	296	60	134
	Total	335	74	142
Actual traffic flow	EV_vehicle	36	13	18
	GAS_vehicle	299	61	132
	Total	335	74	142
**Precision**	EV_vehicle	0.923	0.929	0.867
**Recall**	EV_vehicle	1	1	0.722

## 5. Conclusion and future work

We propose an New Energy Vehicle Recognition and Traffic Flow Statistics approach (NEVTS). The approach is easily and efficiently constructed and can be migrated to other related applications without losing its generality. Extensive experiments are performed on real datasets. The results demonstrate that the NEVTS approach satisfies both real-time and accuracy requirements. One potential limitation of our proposed method is that the accuracy of license plate recognition may decrease significantly under exposure to intense sunlight or license plate blocking and late at night, as even humans may have difficulty discerning license plate colors under such conditions, but vehicles can still be detected. Therefore, the detection problem in these scenarios needs to be further solved.

## Data availability statement

The original contributions presented in the study are included in the article/supplementary material, further inquiries can be directed to the corresponding author.

## Author contributions

YH and MK proposed the conceptualization thought and proposed the methodology presented in this manuscript. YH wrote the main manuscript text. YH and ZS conducted the investigation and obtained the necessary resources for the experiments. YH and MZ verified the method in the manuscript. All authors reviewed the manuscript. All authors contributed to the article and approved the submitted version.
